# Case Report: *De novo DHX37* mutations in Saudi patients with 46,XY differences of sex development

**DOI:** 10.3389/fendo.2025.1622036

**Published:** 2025-07-22

**Authors:** Abeer Alabduljabbar, Sara Abid, Dania Farooq, Sara Aljazaeri, Yara Khamag, Raghad Alhuthil, Latifah Alfahad, Afaf Alsagheir

**Affiliations:** ^1^ Department of Pediatrics, King Faisal Specialist Hospital and Research Centre, Riyadh, Saudi Arabia; ^2^ College of Medicine, Alfaisal University, Riyadh, Saudi Arabia; ^3^ Department of Radiology, King Faisal Specialist Hospital and Research Centre, Riyadh, Saudi Arabia

**Keywords:** DHX37, gonadal dysgenesis, testicular regression syndrome, ambiguous genitalia, genetic testing, Saudi Arabia

## Abstract

Differences of sex development (DSD) are a group of congenital conditions involving atypical chromosomal, gonadal, or anatomical sex development. *DHX37*, a gene involved in ribosome biogenesis, located on chromosome 12, at the 12q24.31 region, has recently emerged as a contributor to 46,XY DSD, particularly gonadal dysgenesis and testicular regression syndrome (TRS). This study presents a case series from Saudi Arabia highlighting novel and known *DHX37* variants in three patients with 46,XY DSD. Three Saudi patients presented with ambiguous genitalia, non-palpable or atrophic testes, and hypergonadotropic hypogonadism. Identified variants included two known (p.Arg308Gln, p.Arg674Trp) and one novel (p.Gly478Val) missense mutation. Phenotypic variability ranged from complete testicular regression to partial gonadal dysgenesis. Thus, this is the first case series of *DHX37*-related DSD in Saudi Arabia, expanding the mutational spectrum and reinforcing the gene’s role in testicular development. Genetic testing, particularly whole-exome sequencing, is essential for accurate diagnosis and management, especially in regions with high consanguinity.

## 1 Introduction

Differences of sex development (DSD) encompass a group of congenital conditions characterized by atypical development of chromosomal, gonadal, or anatomical sex (1). Among these, individuals with a 46 XY karyotype may present with varying degrees of undervirilization, including ambiguous genitalia or incomplete masculinization (2). This spectrum includes complete and partial gonadal dysgenesis (CGD, PGD) and testicular regression syndrome (TRS) (3, 4).

TRS is a rare subtype of 46,XY DSD marked by partial or complete absence of testicular tissue in one or both gonads, despite the presence of the 46,XY (5). It affects approximately 1 in 20,000 boys and is often attributed to prenatal testicular loss, possibly due to vascular accidents (6). Diagnosis typically requires a multidisciplinary approach involving clinical evaluation, hormonal profiling, imaging, cytogenetic testing, and, in some cases, surgical exploration (6).

The genetic basis of 46,XY DSD is becoming increasingly well understood, with several genes now recognized as key regulators of testicular differentiation and gonadal development. Among these, DEAH-box RNA helicase (*DHX37)* has emerged as a notable contributor, particularly in cases of 46,XY gonadal dysgenesis and TRS. Initially, *DHX37* mutations, located on chromosome 12, at the 12q24.31 region (7), were reported in syndromic neurodevelopmental disorders involving microcephaly, global developmental delay, cardiac and renal anomalies, and seizures (8–10). However, more recent findings have demonstrated its role in non-syndromic DSD, with pathogenic heterozygous missense variants linked to a wide phenotypic spectrum—from phenotypic females to males with undescended or atrophic testes (10, 11).

Currently, 21 distinct *DHX37* variants have been reported in 58 individuals with 46,XY DSD, spanning a wide phenotypic spectrum—from phenotypic females to males with undescended or atrophic testes (12). The gene plays a central role in ribosome biogenesis, and its mutations are thought to specifically impair gonadal development without broader systemic involvement (8, 9).

Here, we present a case series of three patients with 46,XY DSD from a tertiary care center in Saudi Arabia, each carrying heterozygous *DHX37* missense variants, including one novel *de-novo* mutation (p.Gly478Val). This report expands the genotypic and phenotypic spectrum of *DHX37*-related DSD and underscores the value of genetic testing in elucidating the etiology of 46,XY DSD—particularly in populations with high consanguinity, such as Saudi Arabia, where the prevalence of rare genetic disorders is elevated (13).

## 2 Methodology

Over the past 10 years (2014–2024), our institution’s endocrinology clinic evaluated 500 patients with various types of DSD. Among them, genetic testing identified three individuals with pathogenic variants in the *DHX37* gene associated with 46,XY DSD.

Genetic testing was conducted as part of routine diagnostic evaluation for all three patients. Genomic DNA was extracted from peripheral blood samples, and whole-exome sequencing (WES) was performed using the Illumina HiSeq2500 platform with an average target depth of 80×. DNA libraries were prepared using the Agilent SureSelect All Exons V6 (50 Mb) capture kit.

Identified variants were assessed and classified according to the American College of Medical Genetics and Genomics (ACMG) guidelines (14), using the Varsome database (15).

**Figure 1:**
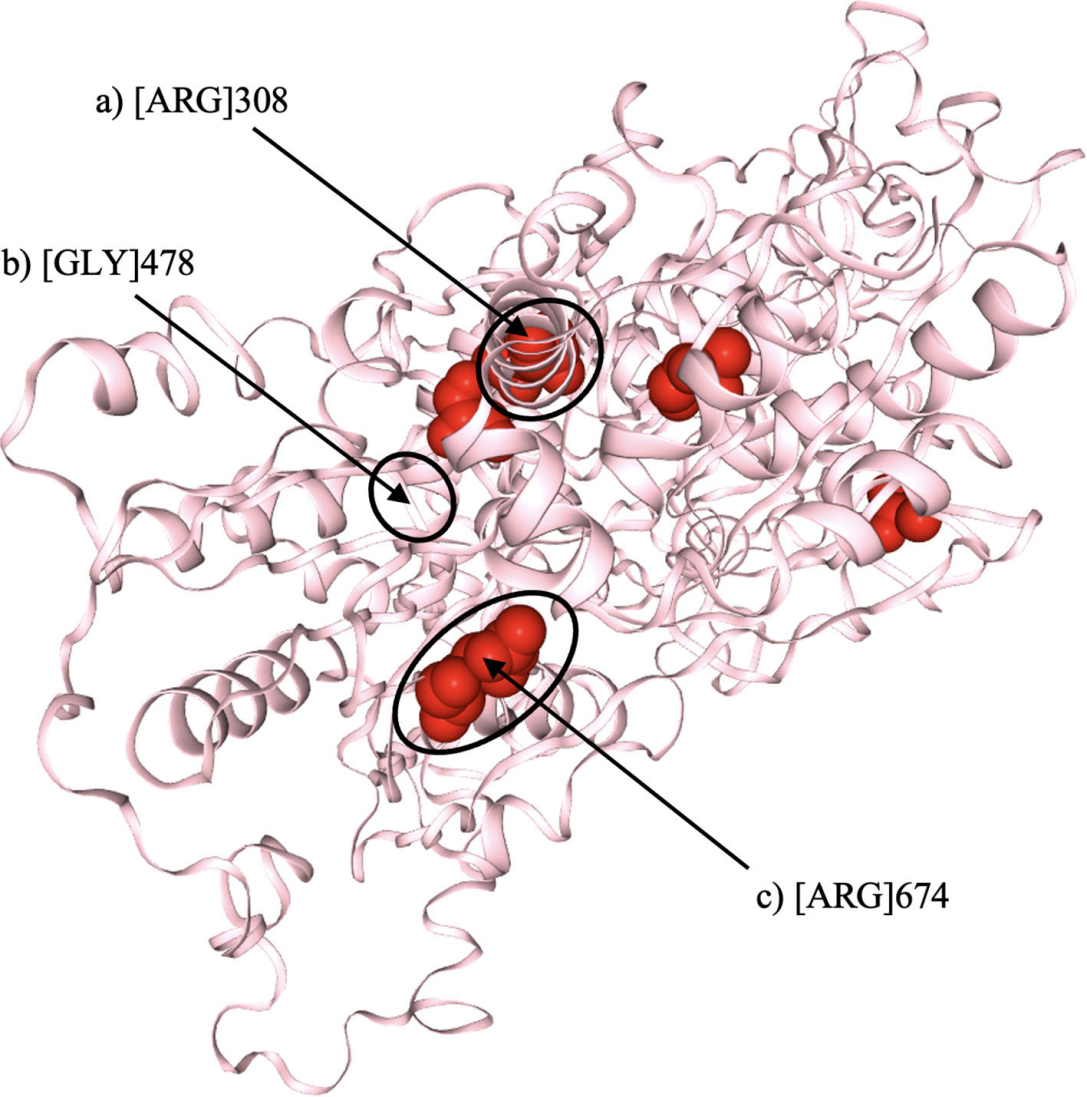
Three-dimensional structural model of the *DHX37* protein, illustrating the locations of the three identified variants (indicated by arrows). **(a)** Substitution of arginine (Arg) at position 308 with glutamine (p.Arg308Gln). **(b)** Substitution of glycine (Gly) at position 478 with valine (p.Gly478Val). **(c)** Substitution of arginine (Arg) at position 674 with tryptophan (p.Arg674Trp). All three variants are predicted to disrupt hydrogen bonding within conserved helicase domains, potentially impairing protein function. Structural modelling was performed using SwissModel (PDB ID: 7d4i, chain RZ).

To further investigate the structural impact of the identified mutations, *in-silico* protein modeling was conducted using SwissModel, based on Protein Data Bank (PDB) entry 7d4i, chain RZ (**Figure 1**) (16).

Ethical approval was obtained from the ethics committee at KFSHRC (reference: 2231110), and written consent was taken from all patients’ legal guardians.

## 3 Results

A summary of clinical, biochemical, and genetic features is presented in **Tables 1**, **2**.

**Table 1:** Clinical characteristics of patients with *DHX37* mutation.

Feature	Patient 1	Patient 2	Patient 3
Age of presentation and the age of hormonal testing	2 years	9 years	2 years
Assigned gender at birth	Female	Male	Male
Karyotype	46,XY	46,XY	46,XY
Gonadal palpability	Non-palpable	Non-palpable	Non-palpable
Phenotype	TRS	TRS	PGD
Genital findings	Phallus-like, posterior labial fusion, single opening	Micropenis, fused labioscrotal folds, single opening	Phallus 0.5 cm, fused labioscrotal folds, single opening
Imaging findings	Absence of both testicular tissue or only atrophic gonadal remnants	Absence of functional testicular tissue with only remnants present	Presence of a structure resembling testicular tissue in the inguinal region
Family history	Negative	Negative	Positive (maternal uncles)
FSH (IU/L) *(Ref: <0.1–7.1)*	96.8	59.2	54.2
LH (IU/L) *(Ref: <0.1–4.0)*	5.0	25.2	23
Androstenedione (nmol/l) *(Ref: 1.0–2.4)*	< 1	< 1	< 1
Testosterone (nmol/l) *(Ref: 0.1–1.12)*	< 0.10	< 0.10	< 0.10
DHT (pg/ml) *(Ref: ≤50)*	< 50	< 50	< 50
DHEA (ng/ml) *(Ref: <2.9)*	< 0.5	< 0.5	< 0.5
Sex assignment	Reassigned to male	Continued male	Continued male
Surgical intervention	Surgical correction, orchidopexy	Bilateral orchiectomy	Orchidopexy
Hormonal therapy	GH + Testosterone	Testosterone	Testosterone
Age at last follow-up	15 years	13 years	13 years

TRS, testicular regression syndrome; PGD, partial gonadal dysgenesis, Ref., reference range; FSH, follicle-stimulating hormone; LH, luteinizing hormone; DHT, dihydrotestosterone; DHEA, dehydroepiandrosterone; GH, growth hormone.

**Table 2:** Heterozygous variants in the *DHX37* gene (Transcript: NM_032656.4) identified in three patients with 46,XY DSD.

Case	DNA change	Protein change	Exon	dbSNP ID	Population frequency	Conservation	*In-silico* prediction	ACMG classification	Inheritance
1	c.923G > A	p.Arg308Gln	6	rs1384892917	Extremely rare (< 0.0001)	Highly conserved across species	SIFT: Deleterious PolyPhen-2: Probably damaging Mutation Taster: Disease causing	Pathogenic (17)	Heterozygous, *de novo* (AD)
2	c.2020C > T	p.Arg674Trp	15	rs1954336272	Absent in gnomAD	Highly conserved (arginine critical in helicase domain)	SIFT: Deleterious PolyPhen-2: Probably damaging REVEL: 0.94 (likely pathogenic)	Likely pathogenic (18)	Heterozygous, *de novo* (AD)
3	c.1433G > T	p.Gly478Val	11	Not reported (novel)	Not in gnomAD/dbSNP	Highly conserved glycine residue	SIFT: Deleterious PolyPhen-2: Possibly damaging MutationTaster: Disease causing REVEL: 0.78 (likely pathogenic threshold >0.5)	VUS	Heterozygous, *de novo* (AD)

ACMG, American College of Medical Genetics and Genomics; VUS, variant of uncertain significance; AD, Autosomal Dominant; REVEL, Rare Exome Variant Ensemble Learner (*in-silico* pathogenicity prediction tool); SIFT, Sorting Intolerant From Tolerant (*in-silico* tool), gnomAD, genome aggregation database

### 3.1 Patient 1

A 2-year-old, initially raised as female, was referred for evaluation of ambiguous genitalia. Examination revealed a phallus-like structure, posterior labial fusion, and a single perineal opening. The karyotype was 46,XY with a positive *SRY* gene. Hormonal evaluation showed hypergonadotropic hypogonadism and poor androgen response to hCG stimulation, suggesting primary gonadal failure.

**Figure 2:**
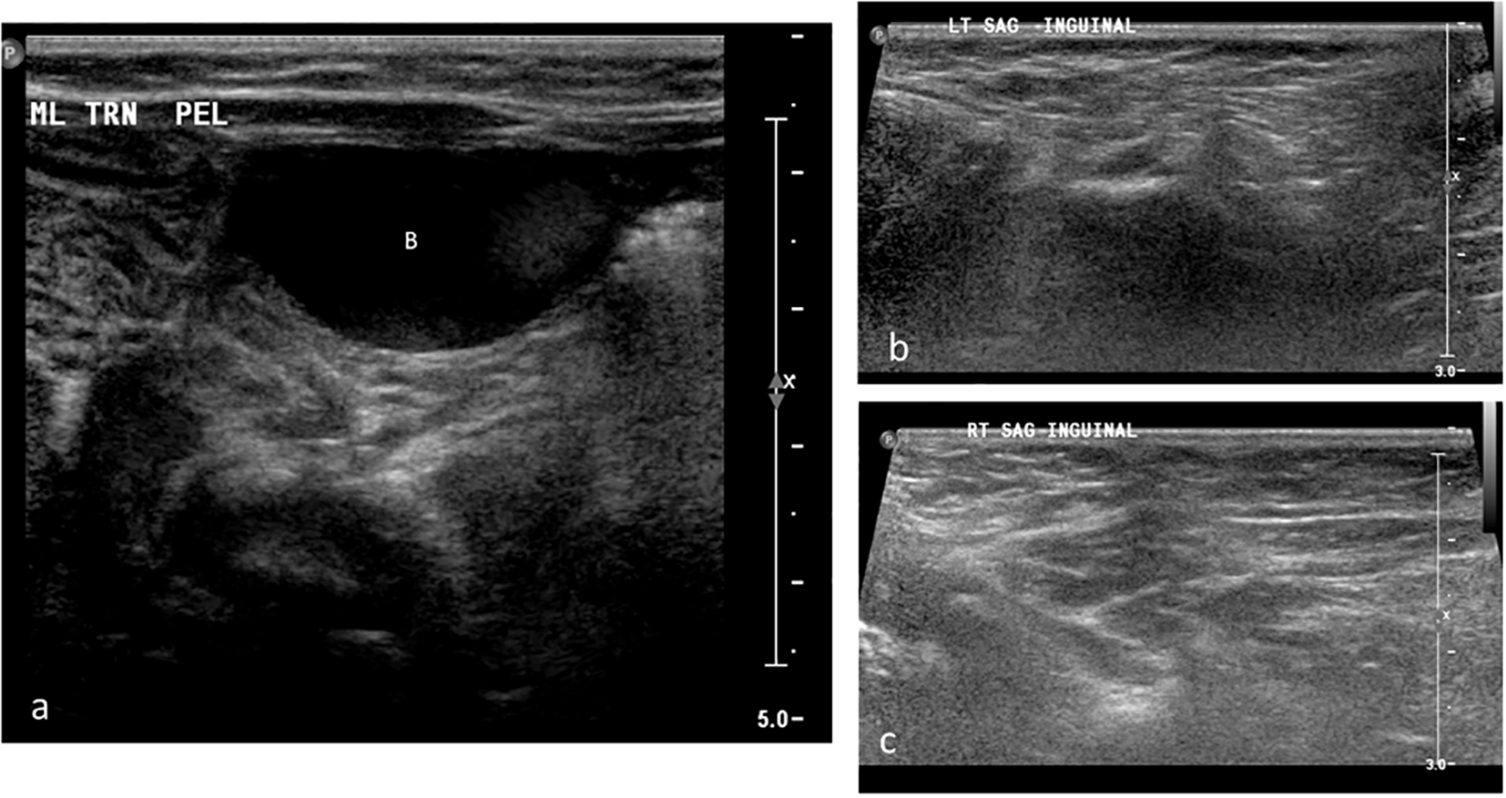
Grayscale ultrasound images of the pelvis and bilateral inguinal canals in Patient 1. **(a)** Transverse pelvic scan showing the urinary bladder (B); no uterus or gonadal structures are visualized posterior to the bladder. **(b)** Longitudinal scan of the left inguinal canal demonstrating absence of gonadal tissue (arrow). **(c)** Longitudinal scan of the right inguinal canal also showing no visible gonadal tissue.

**Figure 3:**
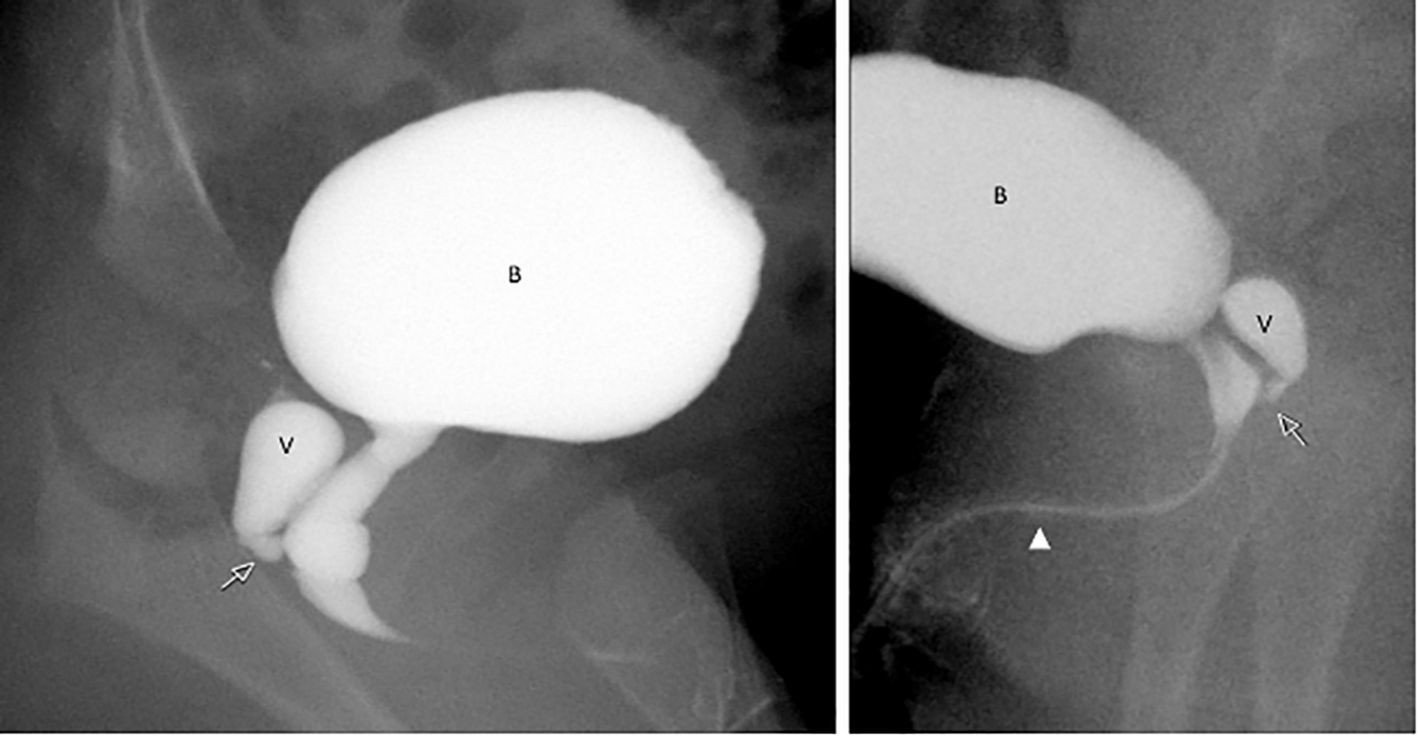
Catheter genitogram images from Patient 1 illustrating internal urogenital anatomy. The contrast study shows a vaginal remnant (V) posterior to the urinary bladder (B), joining the elongated urethra at the level of the urogenital sinus (arrow). The urethra is elongated and male like in configuration (arrowhead), consistent with undervirilized external genitalia.

**Figure 4:**
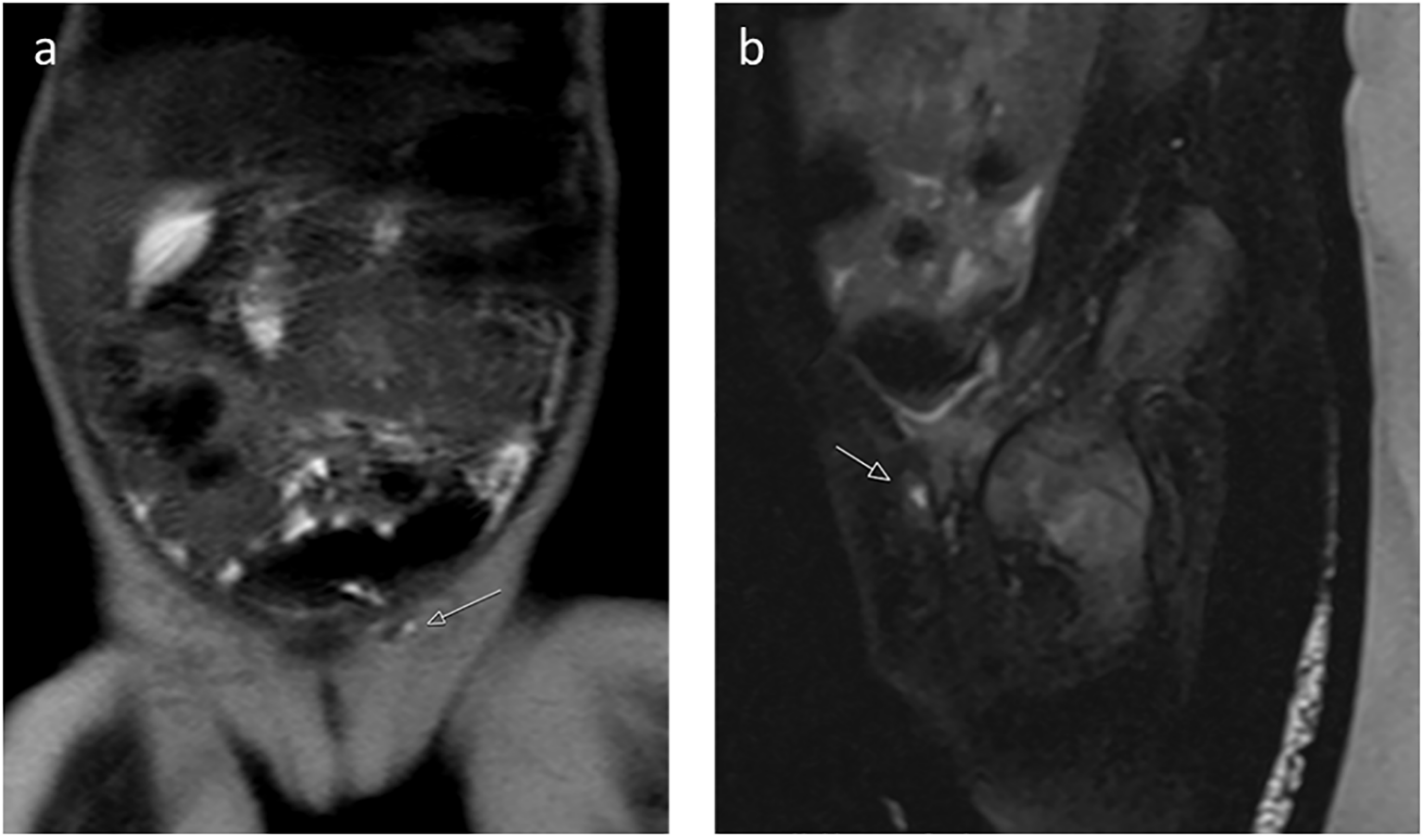
**(a)** Coronal T2-weighted image and **(b)** sagittal T2-weighted image with fat saturation reveal a small, high-signal intensity structure in the left inguinal canal, suggestive of undescended gonadal tissue, and showed a male-type urethra with a small vaginal-like structure connecting to the urogenital sinus (Patient 1).

Imaging revealed no uterus or ovaries. Ultrasound showed absence of testicular tissue (**Figure 2**). A genitogram demonstrated an elongated urethra with posterior outpouching (**Figure 3**), and an MRI showed a male-type urethra with a small vaginal-like structure connecting to the urogenital sinus with a small, high-signal intensity structure in the left inguinal canal, suggestive of undescended gonadal tissue (**Figure 4**). Cystoscopy showed a prostatic utricle and a single introitus. Laparoscopy identified a malformed right intra-abdominal testis and a left testis near the internal inguinal ring.

At 3 years of age, the patient was assessed for short stature, with a recorded height of 90.1 cm (–2.64 SD). Growth hormone stimulation testing using clonidine and glucagon yielded a peak GH level of 8.6 ng/ml, leading to the initiation of growth hormone therapy, which resulted in a 10 cm/year growth velocity during the first year. In the second year, growth velocity was 8 cm/year, followed by a sustained rate of 5–6 cm/year in subsequent years. At age 13 years, whole exome sequencing identified a *de-novo* heterozygous pathogenic *DHX37* variant (c.923G > A:p.Arg308Gln). This patient was diagnosed with TRS. Following a multidisciplinary team evaluation—including pediatric endocrinology, psychology, urology, and ethics consultation—the family received comprehensive counseling on the underlying diagnosis, prognosis, and available management options, including potential gender identity development, fertility implications, and surgical outcomes. After several sessions and with psychological support, the family elected to proceed with male gender assignment. The patient was initiated on testosterone replacement therapy to promote virilization. Subsequently, he underwent staged corrective genital surgery, including hypospadias repair and scrotoplasty. Postoperatively, he showed good surgical outcomes, with appropriate penile appearance and urinary function. At the last follow-up, both the patient and family expressed satisfaction with the decision and treatment outcomes, and the patient has shown stable psychosocial adaptation in the male gender role.

### 3.2 Patient 2

A 9-year-old child, raised as male, presented with ambiguous genitalia; otherwise healthy, his karyotype analysis revealed a 46,XY chromosomal pattern. Physical examination showed a small phallus, fused labioscrotal folds, and non-palpable gonads. Hormonal evaluation demonstrated low baseline testosterone and gonadotropin levels, with no significant response to human chorionic gonadotropin (hCG) stimulation testing. Pelvic imaging did not reveal müllerian structures or definitive testicular tissue but identified a prostatic utricle and structures suggestive of atrophic testes.

Laparoscopy confirmed the presence of bilateral intra-abdominal atrophic testicular remnants, consistent with TRS. Genetic testing identified a *de-novo* heterozygous likely pathogenic *DHX37* variant (c.2020C > T:p.Arg674Trp), further supporting the diagnosis. He received four doses of testosterone at the age of 9 years old. After the testosterone injection, the patient started to have a hoarse voice, he got taller, and he started to have body, pubic, and armpit hair. Given the non-functional nature of the intra-abdominal gonadal remnants and the increased risk of malignant transformation, a decision was made to proceed with bilateral orchiectomy at 11 years of age. The patient continued to be assigned and raised as male, with ongoing psychological support and endocrine follow-up to address pubertal development and long-term care. At the most recent follow-up, he appeared clinically well and vitally stable. Examination revealed pubic hair, a small stretched phallus measuring 1 cm, a single perineal opening, fused labioscrotal folds resembling a scrotum, and an absence of palpable testes. No additional health concerns were reported.

### 3.3 Patient 3

**Figure 5:**
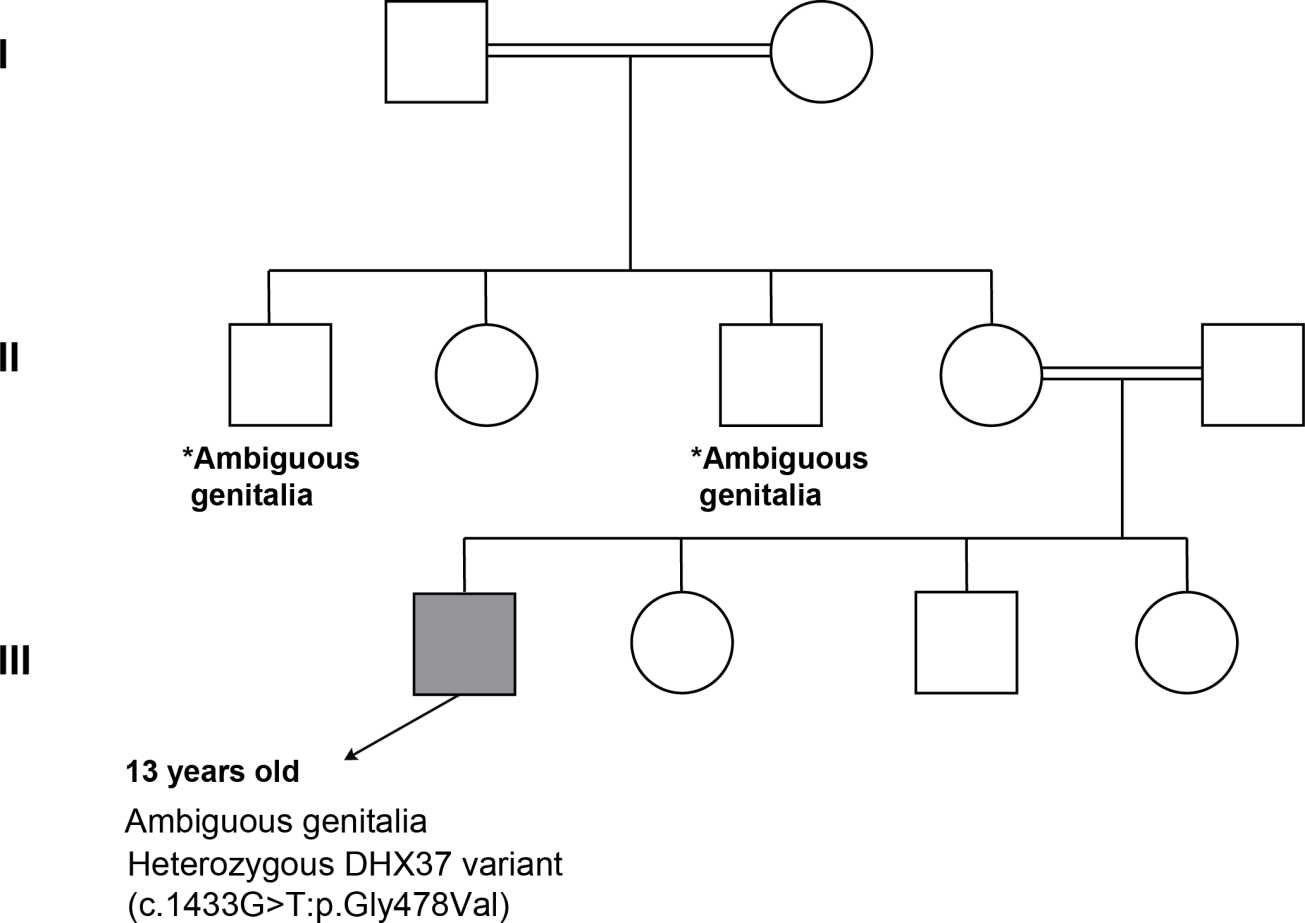
Pedigree analysis of family of Patient 3. *Both maternal uncles had ambiguous genitalia (no identified etiology, segregation analysis was done for both parents and was negative).

A 2-year-old male presented with ambiguous genitalia, primary gonadal failure, and a positive family history of similar cases in two maternal uncles (**Figure 5**), although further details regarding their conditions were unavailable. Physical examination revealed a micropenis, fused labioscrotal folds, and a single perineal opening. Laboratory evaluation at 2 years of age showed elevated gonadotropin levels, and human chorionic gonadotropin (hCG) stimulation testing demonstrated a poor androgen response, consistent with primary gonadal failure. Imaging studies identified an inguinal structure consistent with testicular tissue.

At the initial presentation to our clinic, the patient underwent biochemical evaluation followed by genetic testing, which initially yielded negative results. Initially, the patient was diagnosed with partial gonadal dysgenesis at the age of 2 years. After multidisciplinary discussions with the family, a decision was made to raise the patient as a male and assign a male gender. By the age of 3 years, he received testosterone to enhance phallic growth, and by the age of 4 years, he underwent bilateral orchidopexy. However, based on strong clinical suspicion of an underlying genetic etiology, a repeated genetic test identified a novel variant of uncertain significance (VUS), the *DHX37* variant (c.1433G > T; p.Gly478Val). Parental testing confirmed the absence of the variant, supporting its *de-novo* origin. He is currently under regular follow-up. Testosterone replacement therapy is planned at the appropriate time to induce pubertal development. The patient is reported to be doing well, attending school regularly, and has no additional health concerns. The patient remains under regular follow-up to monitor development and assess the potential risk of gonadal malignancy.

## 4 Discussion

In this case series, we identified three Saudi patients with heterozygous missense variants in *DHX37*: p.Arg308Gln, p.Arg674Trp, and a novel variant, p.Gly478Val. All variants affect highly conserved residues within functional domains, likely altering helicase activity and interactions with RNA or associated cofactors essential for ribosome assembly (9). Each patient had a 46,XY karyotype and presented with ambiguous genitalia, non-palpable or atrophic testes, and hypergonadotropic hypogonadism—consistent features of underlying gonadal dysfunction.

This is the first report of *DHX37*-related DSD in the Saudi population, expanding the gene’s mutational spectrum and emphasizing the importance of population-specific genetic studies (9). With the high rates of consanguinity in the region (13), investigating such rare variants is critical for improving diagnostic accuracy and informing genetic counseling.

None of the patients exhibited intellectual or developmental delay, aligning with previous findings that *DHX37*-related DSD is typically non-syndromic (9). Patient 1 carried the c.923G > A (p.Arg308Gln) variant, classified as pathogenic (17), and repeatedly associated with TRS as reported previously in five affected patients that had gonadal dysgenesis phenotype (7). Patient 2 had the p.Arg674Trp variant, which is classified as likely pathogenic (18), and affects a conserved residue and likely compromises helicase function (7). Patient 3 was found to carry a novel p.Gly478Val variant. Although it was classified as VUS according to ACMG guidelines (14) in the laboratory report, the patient was clinically diagnosed with PGD.

Phenotypic variability among *DHX37* mutation carriers is well documented and was evident in our series. While Patient 1 presented with complete gonadal regression, Patient 3 showed partial dysgenesis with some residual testicular tissue. This variability supports the hypothesis that additional genetic modifiers, epigenetic influences, or environmental factors may contribute to disease expression (8). Furthermore, the family history of genital ambiguity in Patient 3 raises the possibility of germline mosaicism, a phenomenon previously described in other DSD-associated gene (19, 20).

These cases highlight the clinical utility of WES in diagnosing DSD, particularly when hormonal and imaging studies are inconclusive or yield overlapping findings. In Patient 1, genetic confirmation of a *DHX37* mutation supported gender reassignment and initiation of testosterone therapy. In Patient 2, the identification of a pathogenic *DHX37* variant contributed to the decision for bilateral orchiectomy due to the presence of non-functional intra-abdominal gonadal remnants and associated malignancy risk. In contrast, Patient 3 underwent orchidopexy and testosterone therapy prior to genetic testing. The molecular diagnosis did not directly influence initial management; it retrospectively confirmed the clinical diagnosis of PGD, although earlier genetic testing could have significantly enhanced the diagnostic process by integrating with clinical and biochemical findings, thereby enabling the multidisciplinary team to make more informed decisions regarding patient management. It would also have supported more accurate genetic counseling, helped guide future care plans, and informed discussions with the family regarding recurrence risk in future pregnancies.

Overall, these findings underscore the growing importance of early genetic testing in DSD evaluation, not only for precise diagnosis but also for anticipating long-term endocrine, surgical, and psychosocial management needs. Given the phenotypic overlap between *DHX37*-related DSD and mutations in other genes such as *NR5A1* and *MAP3K1*, WES serves as a valuable tool to guide individualized care (21).

Functionally, *DHX37* may influence testicular development by modulating the WNT signaling pathway, which is dosage-sensitive and essential for gonadal differentiation. Disruptions at critical developmental stages may lead to a spectrum of outcomes, from partial dysgenesis to complete regression. Zidoune et al. reported the only known homozygous *DHX37* mutation (p.T477M) in a French TRS patient. Interestingly, the patient’s father—although fertile—had unilateral testicular agenesis, supporting autosomal dominant inheritance with high penetrance (22).

A review by Peng et al. identified 60 patients with *DHX37*-related 46,XY DSD, with p.Arg308Gln and p.Arg674Trp being the two most common variants, accounting for 36.67% and 11.67% of cases, respectively. The detection frequency of *DHX37* mutations varies across DSD cohorts (0.77%–45.45%) and is particularly high in TRS and 46,XY gonadal dysgenesis subgroups (23). Notably, 46,XX female carriers appear unaffected in terms of gonadal development and fertility, while incomplete penetrance is observed in 46,XY males (23).

The management primarily involves surgical intervention and hormone replacement therapy at appropriate developmental stages. However, the long-term prognosis remains uncertain. Although the molecular mechanisms are not yet fully understood, alterations in ribosome synthesis, cell cycle regulation, NF-kB, and WNT signaling have been implicated (23).

A limitation of our study is the lack of functional validation for the novel p.Gly478Val variant. Future *in-vitro* or *in-vivo* studies are needed to determine its impact on RNA helicase activity and ribosome biogenesis. Long-term follow-up will also be essential to monitor pubertal progression and fertility outcomes, particularly in patients undergoing hormonal or surgical intervention.

In conclusion, our case series contributes to the growing evidence supporting *DHX37* as a key gene implicated in 46,XY DSD. The identification of a novel variant in a Saudi patient expands the known mutational landscape and reinforces the importance of genetic testing for ambiguous genitalia, particularly in populations with high consanguinity. Ongoing research is needed to elucidate the molecular mechanisms by which *DHX37* mutations impair gonadal development and to identify potential therapeutic targets.

## Data Availability

The raw data supporting the conclusions of this article will be made available by the authors, without undue reservation.

## References

[1] Lee PA Houk CP Ahmed SF Hughes IA . International Consensus Conference on Intersex. Consensus statement on management of intersex disorders. Pediatrics. (2006) 118:e488–500. doi: 10.1542/peds.2006-0738, PMID: 16882788

[2] Lee PA Nordenström A Houk CP Ahmed SF Auchus R Baratz A . Global disorders of sex development update since 2006: perceptions, approach and care. Horm Res Paediatr. (2016) 85:158–80. doi: 10.1159/000442975, PMID: 26820577

[3] El Beck MD Germano CW Barros BA Andrade JG Guaragna-Filho G Paula GB . Why pediatricians need to know the disorders of sex development: experience of 709 cases in a specialized service. J Pediatr (Rio J). (2020) 96:607–13. doi: 10.1016/j.jped.2019.04.007, PMID: 31254527 PMC9432188

[4] Bashamboo A McElreavey K . Mechanism of sex determination in humans: insights from disorders of sex development. Sex Dev. (2016) 10:313–25. doi: 10.1159/000452637, PMID: 27915330

[5] Hegarty PK Mushtaq I Sebire NJ . Natural history of testicular regression syndrome and consequences for clinical management. J Pediatr Urol. (2007) 3:206–8. doi: 10.1016/j.jpurol.2006.08.007, PMID: 18947736

[6] Heksch RA Matheson MA Tishelman AC Swartz JM Jayanthi VR Diamond DA . Testicular regression syndrome: practice variation in diagnosis and management. Endocr Pract. (2019) 25:779–86. doi: 10.4158/EP-2019-0032, PMID: 31013155

[7] da Silva TE Gomes NL Lerário AM Keegan CE Nishi MY Carvalho FM . Genetic evidence of the association of DEAH-box helicase 37 defects with 46,XY gonadal dysgenesis spectrum. J Clin Endocrinol Metab. (2019) 104:5923–34. doi: 10.1210/jc.2019-00984, PMID: 31287541

[8] McElreavey K Pailhoux E Bashamboo A . DHX37 and 46,XY DSD: A new ribosomopathy? Sex Dev. (2022) 16:194–206. doi: 10.1159/000522004, PMID: 35835064

[9] McElreavey K Jorgensen A Eozenou C Merel T Bignon-Topalovic J Tan DS . Pathogenic variants in the DEAH-box RNA helicase DHX37 are a frequent cause of 46,XY gonadal dysgenesis and 46,XY testicular regression syndrome. Genet Med. (2020) 22:150–9. doi: 10.1038/s41436-019-0606-y, PMID: 31337883 PMC6944638

[10] de Oliveira FR Mazzola TN de Mello MP Francese-Santos AP Lemos-Marini SH Maciel-Guerra AT . DHX37 and NR5A1 variants identified in patients with 46, XY partial gonadal dysgenesis. Life (Basel). (2023) 13:1093. doi: 10.3390/life13051093, PMID: 37240737 PMC10222664

[11] Kulkarni V Chellasamy SK Dhangar S Ghatanatti J Vundinti BR . Comprehensive molecular analysis identifies eight novel variants in XY females with disorders of sex development. Mol Hum Reprod. (2023) 29:gaad001. doi: 10.1093/molehr/gaad001, PMID: 36617173 PMC10167928

[12] Jiang W Yu J Mao Y Tang Y Cao L Du Q . Identification and functional analysis of a rare variant of gene DHX37 in a patient with 46, XY disorders of sex development. Mol Genet Genomic Med. (2024) 12:e2453. doi: 10.1002/mgg3.2453, PMID: 38769888 PMC11106588

[13] Alshamlani LK Alsulaim DS Alabbad RS Alhoshan AA Alkhoder JF Alsaleh NS . Consanguinity and occurrence of monogenic diseases in a single tertiary centre in riyadh, Saudi Arabia: A 2 years cross-sectional study. Appl Clin Genet. (2024) 17:151–8. doi: 10.2147/TACG.S476350, PMID: 39377010 PMC11457763

[14] Richards S Aziz N Bale S Bick D Das S Gastier-Foster J . Standards and guidelines for the interpretation of sequence variants: a joint consensus recommendation of the American College of Medical Genetics and Genomics and the Association for Molecular Pathology. Genet Med. (2015) 17:405–23. doi: 10.1038/gim.2015.30, PMID: 25741868 PMC4544753

[15] Kopanos C Tsiolkas V Kouris A Chapple CE Albarca Aguilera M Meyer R . VarSome: the human genomic variant search engine. Bioinformatics. (2019) 35:1978–80. doi: 10.1093/bioinformatics/bty897, PMID: 30376034 PMC6546127

[16] Ye K . 7d4i.63: swiss-model template library [Internet]. In: Swiss-model. Switzerland (2021) p. 6AD. Available online at: https://swissmodel.expasy.org/templates/7d4i.63.

[17] National Center for Biotechnology Information (NCBI) . ClinVar;. Bethesda (MD: National Library of Medicine (US. (2025) p. Arg308Gln. Available online at: https://www.ncbi.nlm.nih.gov/clinvar/variation/869420/. [VCV000869420.4] NM_032656.4(DHX37):c.923G>A (Accessed May 17, 2025).

[18] National Center for Biotechnology Information (NCBI) . ClinVar. Bethesda (MD: National Library of Medicine (US. (2025) Arg674Trp. Available online at: https://www.ncbi.nlm.nih.gov/clinvar/variation/869421/ (Accessed May 17, 2025).

[19] Nurtjahyo A Huda AN Abadi A Yulisnawati H Fadil P . Disorder of sex development, mosaic genetic disorder 45x, 46xy: A case report. Bioscientia Medicina: J Biomedicine Trans Res. (2022) 6:1393–8. doi: 10.37275/bsm.v6i2.449

[20] Kamel AK El-Ghany A Hoda M Mekkawy MK Makhlouf MM Mazen IM . Sex chromosome mosaicism in the gonads of DSD patients: a karyotype/phenotype correlation. Sexual Dev. (2016) 9:279–88. doi: 10.1159/000442332, PMID: 26656938

[21] Ostrer H . Disorders of sex development (DSD): An update. J Clin Endocrinol Metab. (2014) 99:1503–9. doi: 10.1210/jc.2013-3690, PMID: 24758178

[22] Zidoune H Martinerie L Tan DS Askari M Rezgoune D Ladjouze A . Expanding DSD phenotypes associated with variants in the DEAH-box RNA helicase DHX37. Sex Dev. (2021) 15:244–52. doi: 10.1159/000515924, PMID: 34293745

[23] Peng H Peng W Chen J Hu K Zhang Y Ma Y . Profile of DHX37 gene defects in human genetic diseases: 46, XY disorders of sex development. Front Endocrinol (Lausanne). (2025) 16:1507749. doi: 10.3389/fendo.2025.1507749, PMID: 40026690 PMC11867910

